# Prediction of violence in male schizophrenia using sMRI, based on machine learning algorithms

**DOI:** 10.1186/s12888-022-04331-1

**Published:** 2022-11-01

**Authors:** Tao Yu, Wenzhi Pei, Chunyuan Xu, Xulai Zhang, Chenchen Deng

**Affiliations:** 1 Anhui Mental Health Center; Affiliated Psychological Hospital of Anhui Medical University; Hefei Fourth People’s Hospital; Anhui Clinical Research Center for Mental Disorders, Hefei, 230022 Anhui China; 2 Anhui Province Maternity & Child Health Hospital, Hefei, 230022 Anhui China

**Keywords:** Schizophrenia, Violence, Machine learning, Structural MRI

## Abstract

**Background:**

Violent behavior in patients with schizophrenia (SCZ) is a major social problem. The early identification of SCZ patients with violence can facilitate implementation of targeted intervention.

**Methods:**

A total of 57 male SCZ patients were recruited into this study. The general linear model was utilized to compare differences in structural magnetic resonance imaging (sMRI) including gray matter volume, cortical surface area, and cortical thickness between 30 SCZ patients who had exhibited violence and 27 SCZ patients without a history of violence. Based on machine learning algorithms, the different sMRI features between groups were integrated into the models for prediction of violence in SCZ patients.

**Results:**

After controlling for the whole brain volume and age, the general linear model showed significant reductions in right bankssts thickness, inferior parietal thickness as well as left frontal pole volume in the patients with SCZ and violence relative to those without violence. Among seven machine learning algorithms, Support Vector Machine (SVM) have better performance in differentiating patients with violence from those without violence, with its balanced accuracy and area under curve (AUC) reaching 0.8231 and 0.841, respectively.

**Conclusions:**

Patients with SCZ who had a history of violence displayed reduced cortical thickness and volume in several brain regions. Based on machine learning algorithms, structural MRI features are useful to improve predictive ability of SCZ patients at particular risk of violence.

## Background

Schizophrenia (SCZ) is a debilitating psychiatric disorder, with the prevalence being 1% [1]. Patients with SCZ have been reported to have a heightened risk of exhibiting violent behavior [2, 3]. In comparison with the general population, individuals with SCZ are four to six times more likely to commit violent behavior [4], which can lead to serious harm to others, higher health care costs, and increased stigmatization associated with this disease [5]. Available actuarial assessment tools for violence risk suffer from limited predictive power and the exact mechanisms of violence in SCZ remain unknown, reflecting the difficulty of early detection and intervention of violence in SCZ [6]. Several risk factors associated with violence in SCZ have been well studied, including male sex, substance abuse, as well as childhood trauma, which have been used for development of predictive models based on machine learning method, to identify SCZ patients with the highest risk for violence at the individual level [7–9], but the predictive power of these models is relatively insufficient due to information acquired from phenotypic assessment being limited and violent behavior being heterogeneous in origin [10]. For instance, a previous study by wang et al. established seven predictive models for violence in SCZ patients and found the accuracy and AUC of the optimal model were 62% and 0.63, respectively [11]. Another similar study found the best predictive model achieved an accuracy of 67.8% and an AUC of 0.764 in differentiating patients with violence from those without violence [12]. An article previously published by us showed the predictive model developed by neural net achieved an AUC of 0.667 in predicting violent behavior among male patients diagnosed with SCZ [13]. Hence, there is an urgent need for reliable markers to improve predictive power of models for violent behavior in SCZ.

Structural magnetic resonance imaging (sMRI) as an easy access, high resolution, and non-invasive imaging technique has been widely used to understand the neurobiology of violence in SCZ. Recent sMRI studies have confirmed significant alterations in multiple cerebral regions, in particular frontal and temporal lobes, in SCZ patients with violence [14–17]. For example, a study of cortical morphology showed reduced cortical thickness within the precentral, parietal, temporal, and fusiform cortex in SCZ patients with a history of violence [18]. Kuroki et al. reported significantly smaller temporal lobe volume and insular area in SCZ patients who had a history of serious violent acts than those without a history of violence [19]. A study by Kumari et al. showed lower anterior cingulate volume in SCZ individuals with a history of serious violence when compared to health controls [20]. Another study found that smaller thalamus and amygdala volumes were related with violence [21]. Despite these violence-related brain regions identified at the group level are associated with vulnerability to violent behavior, it is difficult to compute the probability of committing violence at individual level. So far, there are fewer studies using ML with sMRI features to predict violence in SCZ individuals. A study by Gou ningzhi and colleagues used a hybrid machine learning and multimodal neuroimaging data to develop the predictive models for violence in SCZ individuals [22]. Despite the final model based on the combination of gray matter volume, region homogeneity (ReHo) and fractional anisotropy (FA) achieved an accuracy of 90.67%, it is difficult to be implemented in clinical practice due to longer time of operation and higher cost. Besides, only one form of machine learning algorithm was applied in their study. In order to acquire better predictive power, this study combined several machine learning algorithms and structural MRI features to establish prediction models for violence in SCZ.

## Methods

### Study participants

A total of 57 male patients diagnosed with SCZ who fulfilled the International Classification of Disease-10 (ICD-10) diagnostic criteria were recruited from the general psychiatry ward of Hefei Fourth People’ Hospital from July 2021 to December 2021. All participants were divided into violent and non-violent groups based on whether they committed violent behavior or not prior to admission. Violent and non-violent groups included 30 patients with and 27 patients without a history of violence, respectively. All participants were right-handed, no alcohol or substance use disorders, and no neurological diseases. Individuals with head injuries, diagnosed with other psychiatric disorders or contraindicated with MRI were excluded.

#### Definition of violent behavior

Aggressive manifestations in patients with SCZ were evaluated using Modified Overt Aggression Scales (MOAS) which has four subscales (verbal aggression, aggression against objects, physical aggression against oneself, and physical aggression against others) and a five-point rating system (0—4). The weighted total score of MOAS = verbal aggression × 1 + aggression against property × 2 + physical towards self × 3 + physical towards others × 4. Violent behavior was defined by a MOAS weighted total score ≥ 5 [23, 24].

#### sMRI acquisition and post-processing

The structural images of the brain were acquired on the same 3.0 T GE Signa equipped with an eight channel phased array head coil at Hefei Fourth People’ Hospital. The T1-weighted MRI was scanned with the following parameters: repetition time = 8.5 ms; echo time = 3.2 ms; inversion time (TI) = 450 ms; flip angle (FA) = 12°; field of view (FOV) = 256 mm × 256 mm; matrix size = 256 × 256; slice thickness = 1 mm; no gaps; voxel size = 1 mm × 1 mm × 1 mm; 188 sagittal slices; and acquisition time = 296 s. During scanning, all participants were instructed to relax, remain awake with their eyes closed, and move as litter as possible. The earplugs were provided to lessen scanner noise and the sponge pads placed in the coil to minimize head movement. The MRI images of all subjects were checked by an experienced neuro-radiologist and no obvious gross abnormalities were detected. The MRIcron software was used to convert 3DT1WI into NIFIT. The same FreeSurfer software (version 5.3.0) installed in the same Ubuntu Linux version (3.2.0–29-generic) was employed to construct cerebral cortex in this study. The processing procedures produced accurate representations of the cortical surfaces through both intensity and continuity information from the entire three-dimensional MR volume in segmentation and deformation procedures. Recon-all, as one of the core commands of FreeSurfer software, was used to perform the FreeSurfer cortical reconstruction process and to calculate gray matter volume, cortical surface area and cortical thickness of right and left cerebral hemispheres in this study. According to Desikan-Kiliany-Atlas, each cerebral hemisphere was divided into 34 brain regions.

#### Development of predictive models

In the present study, the predictive models were developed using seven machine learning algorithms, including support vector machine (svm), k-nearest neighbor (knn), random forest (rf), generalized linear model net (glmnet), rpart, penalized discriminant analysis (pda), neural network (nnet). A total of 57 male participants were randomly divided into the training and the test sets according to a 1:1 ratio. In order to counteract overfitting, the tenfold cross-validation was conducted in the training set where all subjects were randomly divided into ten equal folds, with nine folds for training the model and the remaining fold serving as validation set. This process was repeated 10 times. Training set is used to automatically adjust the parameters of the model through learning, the validation set for selection of the optimal model and the test set only for evaluation of its performance and generalizability of the final algorithm. To avoid leakage of train/validation/test data set as possible as possible, we have adopted several measures, including deletion of missing variables or values from the entire data, random separation of training and test set, selection of morphology features, no duplicates in the entire data, use of the tenfold cross-validation and so on. In the test set, the performance of each predictive model was assessed using the following metrics: AUC: the value of AUC ranges from 0 to 1; accuracy, sensitivity and specificity. Seven ML algorithms were compared, and the algorithm with highest AUC value was ultimately selected as the optimal model for violence in SCZ. The establishment and performance of predictive models were conducted in R 4.0.5 with package of caret.

### Statistical analysis

The statistical analysis was performed in SPSS 16.0. For demographic information, the continuous variables were expressed as mean (standard deviation) and compared with *t*-test. Moreover, the categorical variables were described using count (percent) and the Chi-Squared Test was used for analysis. The differences in gray matter volume, cortical surface area and cortical thickness between groups were compared by the general linear model after controlling for the whole-brain volume and age. For multiple comparisons correction, false discovery rate (FDR) was utilized. Adjusted *P* < 0.05 was considered to be significantly statistical significance.

## Results

The demographic characteristics of violent and non-violent groups are summarized in Table 1. A significant reduction in the whole-brain volume was observed in patients with a history of violence compared to patients without a history of violence. Patients with violence had a significantly higher age relative to patients without violence. No significant differences were detected in education level and marital status between two groups.

**Table 1:** Comparison of demographic characteristics between groups

Variables	Violent group(30)	Non-violent group(27)	Statistical value	*P* value
Age (year)	39.83 ± 11.03	29.70 ± 8.87	3.793	< 0.001
Whole volume(ml)	1131.6 ± 124.09	1200.2 ± 85.47	-2.404	0.020
Education				
Senior high school or below	25	20	0.363	0.547
Above the senior high school	5	6		
Martial status				
Married/cohabited	19	20	0.759	0.384
Single	11	7		

After controlling for the whole-brain gray matter volume and age, the general linear model showed significant reductions in the thickness of right bankssts and inferior parietal cortex as well as the cortical volume of left frontal pole in the patients with SCZ and violence relative to those without violence (Table 2).

**Table 2:** Different brain regions between groups

Brain regions	Violent group(30)	Non-violent group(27)	F value	*P* value
Right bankssts thickness (mm)	2.2904 ± 0.1478	2.4599 ± 0.1606	-4.149	< 0.001
Right inferiorparietal thickness (mm)	2.2858 ± 0.0933	2.3675 ± 0.0918	-3.328	0.002
Left frontalpole volume (ml)	1009.7 ± 191.01	1201.3 ± 168.26	-4.027	< 0.001

**Fig. 1:**
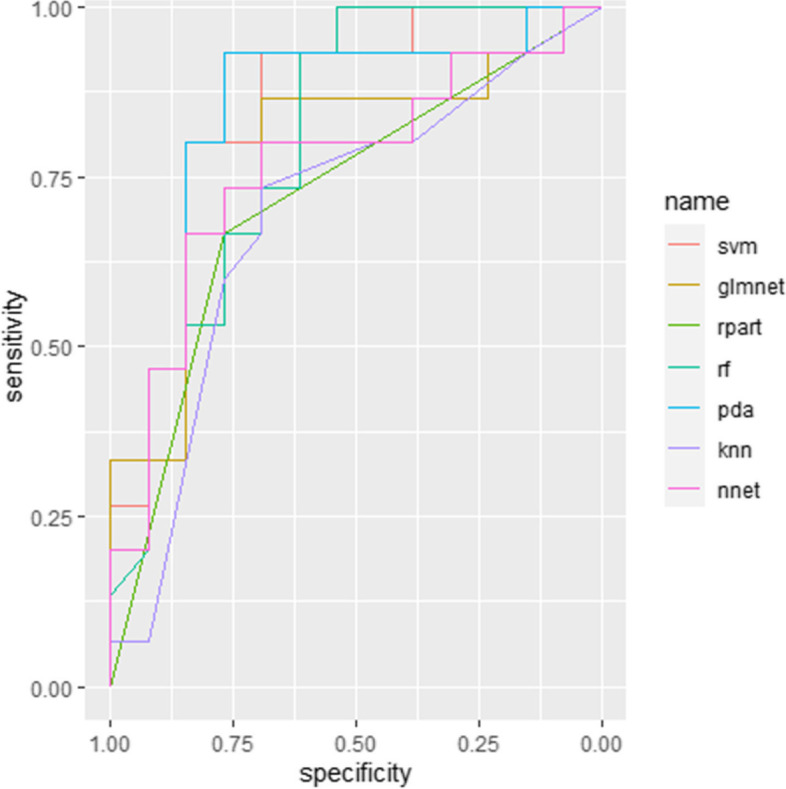
AUC of each machine learning algorithm

These different brain structures between groups including left frontal pole volume as well as right bankssts and inferior parietal cortical thickness were regarded as features for machine learning classification. Among seven predictive models, SVM was identified as the optimal algorithm for violence in SCZ with a balanced accuracy of 0.8231 and an AUC of 0.8410 (Table 3 and Fig. 1).

**Table 3:** The performance of machine learning algorithms

Algorithms	AUC	balanced Accuracy	Kappa	Sensitivity	Specificity
svm	0.8410(0.6826–0.9995)	0.8231	0.6429	0.8000	0.8462
glmnet	0.7692(0.5841–0.9544)	0.7462	0.4948	0.8000	0.6923
rpart	0.7179(0.5463–0.8896)	0.7179	0.4315	0.6667	0.7692
rf	0.8077(0.6384–0.9770)	0.7410	0.4896	0.8667	0.6154
pda	0.8410(0.6760–1.000)	0.8179	0.6392	0.8667	0.7692
knn	0.6923(0.4882–0.8964)	0.6795	0.3571	0.6667	0.6923
nnet	0.7641(0.5781–0.9501)	0.7564	0.5051	0.6667	0.8462

## Discussion

The main findings in the present study were that after controlling for the whole-brain volume and age, the SCZ individuals with a history of violence showed reductions in several brain regions involved in emotion and cognition processing, including left frontal pole volume as well as right bankssts and inferior parietal cortical thickness, compared to those without a history of violence. Subsequently, these different brain structures between groups were used to develop the prediction models for violence among SCZ patients using machine learning method. Ultimately, seven predictive models were established. Through comparing with each other, the SVM had the best performance with a balanced accuracy of 0.8231 and an AUC of 0.841.

In the present study, the finding of reduced whole brain volume in patients with SCZ who had a history of violence, compared to those without a history of violence, is in consistence with results of previous studies [25, 26], suggesting the possibility that certain general cognitive impairments associated with whole brain volume reduction are involved into violence. Besides, we also found abnormalities in several regions implicated in the neuropathology of violence, including left frontal pole, right bankssts and inferior parietal regions. Patients with SCZ who had a history of violent behavior displayed decreased gray matter volume in frontal pole in relation to those without violence, which is consistent with another study [27]. This indicates that changes in the prefrontal cortex including the frontal lobe might be involved in the pathophysiology of violent behavior. The prefrontal cortex is thought to play an important role in executive functioning capacity, including regulation of inhibition, emotions and movement. Damage or dysfunction of this area may interrupt the sending of inhibitory inputs to the limbic system, which is composed of hippocampus and parahippocampal gyrus and increases the risk of unregulated behavior [28], speculating that the region can be regarded as structural markers for violence [29]. Temporal lobe is implicated in emotional processing and its abnormalities are linked to the onset of psychosis, hallucinations and delusions in SCZ [30, 31]. Delusions as one of the important features of SCZ have been most consistently related to violent behavior [32]. In addition, the relation of abnormal temporal lobe with violence is also supported by other evidence that alternations in this region can lead to impaired aggression control and increased impulsivity which belong to aspects of characteristic antisocial personality disorder [30, 33]. We also found SCZ subjects with a history of violence displayed decreased cortex thickness in right parietal lobe as a part of the default-mode network (DMN) which is responsible for self-referential and reflective activity as well as attending to internal and external stimuli in relation to those without violence, in consistent with findings from the previous studies [34, 35]. Taken together, the structure volume of the brain is closely associated with the size, density and arrangement of neurons, and its change may lead to the destruction of circuits in relevant brain regions [35], suggesting abnormal neurodevelopment is vital to the neurobiology of violence in schizophrenia.

In our study, we found SVM was appropriate to structural MRI data and had better predictive performance in differentiating violent from non-violent patients with SCZ than other six machine learning algorithms, with its balanced accuracy and AUC reaching 0.8231 and 0.841, respectively, similar to previous findings that SVM has promising results in neuroimaging [36]. The possible reason is that SVM belongs to one of the machine learning algorithms which can process high-dimensional data and capture nonlinear variable relations. Considering that neuroimaging data are likely to be nonlinear, SVM is able to achieve better performance than other algorithms [37]. To date, studies predicting risk of violence in SCZ patients using neuro-imaging data are sparse. The only research employed multimodal MRI and SVM to identify SCZ patients at high risk of violence. The model based on the single modality of gray matter volume showed an accuracy of 77.33% and an AUC of 0.80 [22]. The possible reason for different predictive power is that our model included more characteristics of cortical morphology, namely cortical surface area, gray matter volume and cortical thickness, to improve the power of recognizing the patients with greater risks of violence. Besides, there have been a few studies which combined machine learning algorithms and demographic and clinical data to differentiate patients with violence from those without violence, but the performance of prediction models was unsatisfactory [11–13]. In this study, the prediction model integrating structural MRI characteristics demonstrated good performance. Above evidence suggests that due to high anatomical resolution of cortical volume, area and thickness, structural MRI features can be suggested to be biologically-based predictive markers.

Several limitations need to be considered. First, our sample size was relatively small, Future studies should recruit more participants to improve the power of predicting violence in SCZ. Second, the inpatients with SCZ enrolled by us were receiving treatment with a medication. Despite effect of medication on cortical morphology is still unknown, the brain structure of SCZ patients might be influenced by antipsychotic treatment. In order to validate our results, future studies should be conducted in first-episode, medication-naive patients with SCZ. Third, the present study lacked external validation, which could limit the generalization of our findings. Future research should perform the external validation in another sample. Fourth, the model developed by male SCZ patients was not applicable to female individuals. Considering difference in brain structure and risk factors between male and female patients, the models based on gender need to be built in future studies.

## Conclusions

In summary, the current study showed difference in cortical morphology between patients with violence and those without violence. Based on these different brain structural regions, seven prediction models were established using machine learning method. SVM had better performance in identifying SCZ individuals who had a history of violence, suggesting based on machine learning algorithms, sMRI features can improve predictive power of models.

## Data Availability

The data that support the findings of this study are available from Hefei Fourth People’ Hospital but restrictions apply to the availability of those data, which were used under license for the current study, and so are not publicly available. Data are however available from the authors upon reasonable request and with permission of Hefei Fourth People’ Hospital.

## Abbreviations

SCZ: Schizophrenia
sMRI: Structural magnetic resonance imaging
MOAS: Modified overt aggression scales
AUC: Area under curve of ROC
ReHo: Region homogeneity
FA: Fractional anisotropy
ICD-10: The International Classification of Disease-10
SVM: Support vector machine
FDR: False discovery rate
ML: Machine learning
